# Sharing clinical information across care settings: the birth of an integrated assessment system

**DOI:** 10.1186/1472-6963-9-71

**Published:** 2009-04-29

**Authors:** Leonard C Gray, Katherine Berg, Brant E Fries, Jean-Claude Henrard, John P Hirdes, Knight Steel, John N Morris

**Affiliations:** 1 The University of Queensland. c/- Academic Unit in Geriatric Medicine, University Department of Medicine, Princess Alexandra Hospital, Brisbane, Australia; 2 University of Toronto, Toronto, Canada; 3 University of Waterloo, Waterloo, Canada; 4 Institute of Gerontology, School of Medicine, University of Michigan, Ann Arbor, USA; 5 Health Systems Research, Veterans Administration Healthcare System, Ann Arbor, Michigan, USA; 6 Versaille University, Paris-Oust Medical School, Paris, France; 7 Department of Health Studies and Gerontology, University of Waterloo, Waterloo, Canada; 8 Homewood Research Institute, Waterloo, Ontario, Canada; 9 Hackensack University Medical Center, New Jersey, USA; 10 Institute for Aging Research, and Alfred A and Gilda Slifka Chair in Social Gerontological Research, Hebrew Senior Life, Boston, Massachusetts, USA

## Abstract

**Background:**

Population ageing, the emergence of chronic illness, and the shift away from institutional care challenge conventional approaches to assessment systems which traditionally are problem and setting specific.

**Methods:**

From 2002, the interRAI research collaborative undertook development of a suite of assessment tools to support assessment and care planning of persons with chronic illness, frailty, disability, or mental health problems across care settings. The suite constitutes an early example of a "third generation" assessment system.

**Results:**

The rationale and development strategy for the suite is described, together with a description of potential applications. To date, ten instruments comprise the suite, each comprising "core" items shared among the majority of instruments and "optional" items that are specific to particular care settings or situations.

**Conclusion:**

This comprehensive suite offers the opportunity for integrated multi-domain assessment, enabling electronic clinical records, data transfer, ease of interpretation and streamlined training.

## Background

The purpose of health care is to provide person-specific rather than site-specific care [1]. With rare exceptions, the site of care is determined by economic considerations and by the structure and policies of the health and welfare systems of each nation. Thus a country's health care structure may stipulate what services are reimbursed at each level of care and thereby effectively preclude their being provided in other locations. Also the availability of informal support systems or lack of them may result in a given location being the site of care for multiple individuals with quite different needs who therefore require strikingly different services. Nonetheless, although certain diseases, levels of acuity and functional deficits may be more common in one location than another and may be required to justify care in a particular site, the specific needs of each individual must be addressed appropriately regardless of that person's location.

Older adults not only use more care, but they receive care in a host of sites, such as hospitals, long term care facilities and even the home. Further, because many older individuals have multiple chronic diseases, the conditions of a particular person that are being addressed in one location often are similar if not identical to those in the site to which the person is next transferred. Rather than targeted to a new disease, the care in the second site usually must address changes in acuity and perhaps the interaction of one or more chronic diseases with a newly discovered condition. Similarly, the transition to inpatient psychiatry from community mental health services for a middle aged adult with schizophrenia is more likely to be driven by a change in the severity of symptoms than the onset of a new type of mental illness.

In industrialized countries, there has been a substantial shift away from institutional models of care of chronic illness, particularly in mental health and disability services. Many governments are now adopting similar approaches to care of older persons. These transformations increase the complexity of the service delivery framework, with multiple service providers often delivering care to individuals, and more frequent changes of care setting occurring, particularly between community and facility settings. Integration and coordination have become central issues in the care of older people, and a variety of demonstration programs have been established in response. [2,3]

These changes demand radical re-rethinking of the organization of clinical information systems that typically have been designed to support single service providers in one setting. Traditional clinical systems tend to focus on a limited set of problems, often reflecting the views of care professionals and funding agencies. Complex, multi-dimensional views of the person seldom come to the fore in such systems. For those who see health and social care from such a perspective, the number and types of meaningful problems is limited, as is the scope of the assessment methodologies and the care they are provided.

With this as backdrop, there is a need for assessment systems and service provision that transcend care settings, enabling the identification of and response to complex needs with person-specific care planning information that can flow with individuals as they move across care settings. By focusing on a limited set of care prerogatives, health care systems often fail to respond adequately to the complex interactions of factors influencing the lives of persons who turn to formal care systems for assistance.

To both improve outcomes and maximize the function of those served, essential health-related information must be transmitted from site to site in a timely manner. The electronic medical record can make such transmission across sites of care easier. However, more than ever, the successful introduction of electronic medical records will depend on the availability of high quality, standardized, clinically relevant data that carry the same meaning independent of the location of care. Moreover, the information must be accessible and useful to diverse stakeholders responding to different issues at various levels of the health care system. Availability of clinical information is one of the key ingredients of successful strategies to better manage chronic illness [4].

Thus, a crucial ingredient of future multi-sector care delivery systems will be a common language of clinical descriptors. This language must be broad in its conceptualization, detailed when necessary, and capable of being interfaced, where possible, with emerging computer-based information technologies.

### The concept of third generation assessment instruments

Many clinical and care services adopt structured approaches to assessment. Typically, these consist of items developed by the organization itself or a panel of assessment tools that attend to aspects of assessment (e.g., the Mini-Mental State Examination for dementia screening, or the Barthel Index for activities of daily living profiling). These systems can be characterized as "first generation" assessment instruments. Their strength is their focus on a specific issue or problem, the establishment of discrete measurement rules, and field testing of the tools for reliability, validity, and utility within a clinical trial environment. Their weakness is the lack of proven utility across different care settings, the focused nature of their assessment, and the difficulty of "cobbling together" a series of what are often lengthy single-purpose tools into a usable, coherent overall assessment battery.

Second generation assessment instruments aim to cross many clinical domains and have applicability in many settings. They also provide a greater opportunity to interpret assessment information systematically to assist in treatment and care planning. This type of instrument requires a broad consensus on the multiplicity of domains to be measured. As with first generation tools, the items must be as relevant as possible to the underlying domains; the only difference is that there is a need for a purposeful trade-off between the number of operational items for any one domain and the overall length of the assessment tool. In a second generation tool, individual items are constructed to record focused information about the individual and it is the assembly of these items into a meaningful set of purposeful domains that form the backbone of a comprehensive assessment schedule. A key attribute of such instruments is the attention to the primary purpose of care planning, i.e., to provide the user with information targeting specific medical, functional, and social problems that need to be addressed and further guidance through care planning protocols. The instrument must then be subjected to extensive field testing to ensure reliability of items and validity of the summary measures that are based on the items. Such a tool has the advantage of providing consistency in item structure; coverage of all relevant care domains; information about the person's needs to guide care planning and the ability to build a robust set of outcome products such as case-mix groupings, quality indicators and eligibility determinants [5].

Third generation instruments extend the concept of second generation tools to multiple care settings. They provide assessment strategies that transcend boundaries between care settings. The focus is on the changing strengths, preferences and needs of the person, rather than the sector which happens to be providing services at a single point in time.

In 2002, the interRAI consortium took up the challenge to refine its array of second-generation assessment tools into a truly integrated third-generation system. The remainder of this paper describes the approach taken and the outcomes of the process to date.

### The interRAI consortium

interRAI is a not-for-profit research consortium of about 50 clinicians, researchers and health administrators from 25 countries. It was established in 1992 with a collective vision that "the assembly of accurate clinical information in a common format within and across services sectors and countries enhances both the well-being of frail persons and the efficient and equitable distribution of resources" [6].

Initially, development efforts focused on long-term residential care, with the Resident Assessment Instrument – Minimum Data Set (RAI-MDS) and its associated case-mix application (Resource Utilization Groups – RUG-III [7]) enjoying wide uptake in nursing homes in North America and Europe. An early version of the instrument was mandated in all Medicare and Medicaid funded nursing homes in the USA in 1990, and has been associated with measurable improvements to the standard of care, particularly when quality indicators derived from the instrument were introduced [8,9]. Other nations that have since adopted the RAI-MDS on a widespread basis include Canada, Finland, Estonia, and Iceland. A series of "case studies" have been published by the Milbank Memorial Fund [10]. Intervention studies suggest that implementation of instruments may influence outcomes [11].

In 1994, a home care version was created – the RAI-HC – which has also enjoyed considerable implementation in several countries [12]. Subsequently, instruments were developed for acute and post-acute care, palliative care, hospital and community mental health, physical and intellectual disability and assisted living. The initial focus of interRAI on care of older persons has since been broadened to address the needs of adults of all ages with complex and disabling physical and mental illnesses.

### interRAI instruments

interRAI instruments are administered by trained assessors who interact with the person, their caregiver(s) and staff (particularly in institutional settings). They also review all available records. It is only after consulting all of these sources of information that "observations" are determined, based on their best judgment if the information is conflicting. For example, in order to determine whether an elderly woman prepares her own meals or performs her own housekeeping in a home care service setting, the assessor will consult the person, her care-giver(s), community service providers, and any available records. The exception is a small number of items that are specifically addressed to the person being assessed, such as aspects of mood and self- reported perception of health. Where there is evidence of cognitive or communication impairment, there will be increased reliance on information provided by care-givers and staff. This approach is complex and requires good judgment on the part of the assessor, but repeatedly has been shown to be reliable. Interviews with persons are therefore conversational in style, and semi-structured.

Once the assessment is completed, scales summarizing major domains (e.g., depression, cognition, and physical function) are calculated. They are not explicitly administered but are computed from the overall observation. The burden of the assessment process is thus minimized. [Copies of interRAI instruments may be obtained by contacting interRAI at . Instruments are copyright, however they are made freely available to non-commercial organizations.]

interRAI instruments have the following design features:

• A fully developed instrument comprises a data set (for clinical data collection), a training manual and a set of algorithms that generate "Clinical Assessment Protocols" (CAPs), scales (including screeners and severity measures), case-mix measures and quality indicators.

• Instruments are designed for application by health professionals trained in the interRAI method with the support of a training manual.

• The instruments primarily inform the clinical assessment and care planning processes – administrative utilities such as planning data, case-mix tools and quality indicators are by-products.

• The instruments have sound psychometric properties, established through extensive field testing and revision of poorly performing items. With moderate levels of training of health professionals (typically 2 or 3 days), high levels of reliability and validity are assured.

• There is a development path for each instrument. Revisions (or upgrades) are produced as clinical practice standards develop. The most mature instrument (for long term residential care of older persons) is now in its fourth version and the home care instrument in its third version.

**Figure 1:**
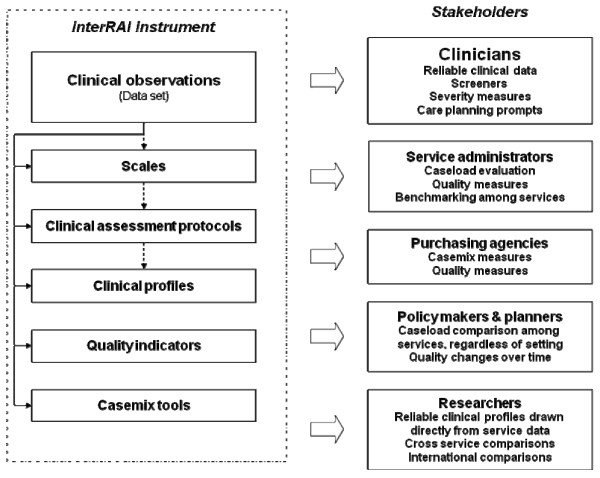
**Schematic representation of an interRAI instrument**.

Thus, interRAI instruments are designed to produce observations and outputs primarily to assist clinicians in assessment and care planning, but have the added advantage of providing an array of purposefully designed information for a wide range of stakeholders. (Figure 1).

There have been many spin-off applications of data from interRAI instruments, including determination of eligibility for services; tracking of individuals' progress over time; structures for resource allocation both within organizations and across nations; rationalizing of services and systems; and projection of need and associated costs [6]. It has been used for cross-national comparisons of needs [13], which contributes to a better understanding of response options related to those needs.

Until 2001, different instruments were designed by different committees comprised of domain experts and measurement scientists from within interRAI and collaborating organizations. In general, the starting point for new instruments was a thorough review of the key issues that were relevant to persons within the care setting for which the instrument was completed. Extensive lists of issues were drawn up, subjected to extensive internal and external review, and only then did the interRAI team begin to "create" the first draft of the instrument. Wherever possible, each team endeavored to use applicable key measures and relevant material from earlier interRAI assessment tools. Thus, when interRAI moved to the RAI-HC, the home care instrument drew heavily on items contained in the nursing home instrument. Where necessary, committees modified items to best suit their care setting. New items were developed, without necessarily referring to items measuring similar concepts in other instruments. As a result, interRAI developed a set of instruments with a similar look and feel, but which in the details did not consistently measure the identical phenomena in the identical way. This was not necessarily a problem if one focused only on care in a single setting; however, it fell short of the full potential of the family of instruments to function as a system linking multiple sectors together.

Recognition of this inconsistency, coupled with an emerging vision of an integrated health information system – one that permitted seamless tracking of persons in multiple service settings – led to the establishment of a process to create the third-generation interRAI suite of instruments.

### The instrument suite concept

The concept of a "suite" of compatible assessment tools arose from the following six considerations:

1) People with chronic illness and disability often move between care settings. There is usually a need to transfer clinical information about the person. If, at the time of transfer, the person's condition or circumstances are stable, the information from the most recent assessment would be current, with the potential to reduce the assessment effort by the receiving agency. In some instances it might eliminate the need completely.

More often, the transfer is associated with a change in status. For example, when there is an admission to hospital there may be changes in functional status associated with a new disabling illness. Similarly, when a person is admitted to a long term care facility or psychiatric hospital from the community, there may be changes in mood and behavior. In this circumstance, prior information around the person's status would provide valuable insights regarding his/her current needs. If a person has a progressive or fluctuating condition, and is moving between care settings, monitoring of change over time would assist clinicians to assess stability and rate of progression.

2) Consistent recording of information across care settings might assist clinicians in other ways: Familiarity with the language, definitions and interpretation of an instrument utilized in the health professionals "home" care setting would facilitate interpretation of a companion instrument performed in another setting. For example, if a measure of ADL dependency is constructed and scored in the same manner in all settings, interpretation of the level of dependency will be simplified. Thus, when a person is discharged from a post-acute service to a home care program, the admitting assessor would quickly be able to appraise the person's needs even before consulting him/her directly.

3) Training of assessors would be simplified. A nurse trained in the use of an instrument in one sector could quickly learn to use a companion instrument in another. Many health professionals work across sectors either concurrently or over the course of their career.

4) There were perceived administrative advantages in addition to those derived from improvements in the efficiency of clinical assessment procedures apparent from the above. In particular, design and construction of software systems to support multi-sector assessment and care planning would be simplified and the availability of common assessment items with common definitions would enable multiple instruments (and new ones) to be built quickly.

5) Comparison of case complexity of persons in different care settings would be facilitated. For example, administrators concerned with the conundrum of relative costs of community and institutional care could compare caseloads against a wide array of measures. interRAI tools have proven extremely successful in comparative analyses of caseloads across similar care settings in different jurisdictions, at local, national and international levels [13,14]. This capacity to compare caseloads would serve to improve equity of access to services.

6) Finally, if instruments are designed to minimize the effect of cultural differences, they provide a powerful capacity to compare the utilization and impact of different health and social systems across nations.

### The development process

In 2001, interRAI established a multinational and multidisciplinary working group of fifteen researchers to create an integrated suite of assessment instruments. During the five year development and testing process the group met for multi-day meetings on thirteen occasions. An estimate of the contributed cost of developing this system already exceeds US$2.5 million. The group continues to meet to support further development of the suite.

The initial challenge to the group was to create a truly integrated suite of assessment tools for long-term institutional care, post-acute care, home care, assisted living, in-patient and out-patient mental health, palliative care and independent living settings, and one that would be applicable worldwide. The first step was to identify a set of domains and associated operational items that were considered mandatory for each new instrument – ***"core items." ***The second task was to create an ***optional ***set of domains and associated items that would appear on several, but not all, instruments in the suite. Finally, there would be ***instrument specific ***domains and items that were applicable on only one or two settings. [Examples of core items are cognitive skills for daily decision -making and mobility. Examples of optional items are certain aspects of memory function or pain. Items related to suicide and dying are specific to mental health and palliative care instruments respectively.]

This task was undertaken by compiling a comprehensive inventory of all existing interRAI items. The inventory was reviewed to identify concepts and items in each instrument – some were shared, others were unique. Among shared items, variations were identified.

Next, the function of items within specific instruments was catalogued. In addition to describing some aspect of a person, item functions included contributions to:

• Clinical Assessment Protocols (CAPs), which are algorithms that suggest care planning actions;

• Case-mix classification systems on which reimbursement formulas were based (e.g., RUG-III for nursing homes, RUG-III/HC for home care, and SCIPP for inpatient psychiatric care);

• Quality indicator systems. In the nursing home RAI this includes the quality measures created by the Zimmerman group, the Morris Mega-QI group, and the National Quality Framework approved quality measures posted on the Centers for Medicare and Medicaid Services website [15]. Quality indicators have also been developed for home care [16], post-acute care [17]), and mental health; [18]

• Commonly-used, standardized interRAI scales (e.g., the Cognitive Performance Scale, and ADL scales [19,20])

Items that served at least one of these purposes were given priority to be selected as ***core ***items.

Committee members solicited and received extensive international feedback from clinicians, researchers and organizations around the world using one or more interRAI instruments. Then items were reviewed and, if necessary, the wording harmonized to ensure consistency across all instruments. Rules for item design were created, and rigorously applied to each item:

• interRAI items are phrased to give clear definition, and they require observations of behaviors or performance over a specific time frame (usually 3 days for the new suite). Item descriptions typically include examples and exclusions to increase the precision of definitions.

• The assessment process follows a usual clinical interview, assimilating information from multiple sources. Clinicians use their professional judgment to appraise diverse information and to assign the most appropriate code to the form.

• The psychometric and distributional properties of items are scrutinized carefully to ensure reliability, validity and clinical relevance.

• Items are not survey questions that are asked precisely the same way and in the same sequence. Rather, the response categories coded by clinicians are the focus of standardization.

• To facilitate applicability to multiple settings the suite refers to "persons" rather than patients, clients or residents.

New instruments were then assembled for each care setting. Core items were considered mandatory and only excluded in exceptional circumstances, where they were clearly not appropriate for a particular setting. Optional items were then considered, and included if considered relevant to the setting. Finally, setting (or instrument) specific items were added.

Extensive international field testing was undertaken. The first five instruments completed were studied: long term care; home care; post-acute care; palliative care and inpatient mental health. Field testing focused on item response rates; inter-rater reliability; and convergent validity of scales within each instrument, typically considering frequently used scales as the benchmark standard. The results are the subject of a separate publication[21], and the reliabilities were closely comparable to previously reported values [22-24].

Subsequently, several other instruments have been added to the suite, using a similar development approach. Examples include new instruments for acute care [25], community mental health, intellectual disability, and assisted living. In addition, new screeners for psychiatry, home care, and emergency department settings were created to be compatible with the full assessments in the suite.

### The outcomes

At the time of writing, ten instruments were complete (Table 1). Several additional instruments are planned for the immediate future.

**Table 1:** The interRAI suite of assessment instruments

Instrument	Item count	Target population
Long term care facility (interRAI LTCF)	257	Residents of nursing homes or chronic hospitals

Assisted living (interRAI AL)	262	Assisted living facilities where residents have light care needs

Acute care (interRAI AC)	96	Frail older patients in acute care hospitals

Post-acute care (interRAI PAC)	214	Rehabilitation and other post-acute inpatients

Home care (interRAI HC)	253	Community based care

Community health assessment (interRAI CHA)	135	Community settings with anticipated light care needs

Palliative care (interRAI PC)	194	Palliative care in community and institutional settings

Mental health (interRAI MH)	304	Mental health inpatients

Community mental health (interRAI CMH)	320	Community mental health services

Intellectual disability (interRAI ID).	287	Persons with intellectual disability in community and facility settings

The instrument data sets range in size from 96 (interRAI AC) to 320 (interRAI CMH). As a general rule, where there was a prior interRAI instrument, the version created for the new suite was notably shorter. The instruments in the new suite share a large proportion of items – a result of the design strategy. The core items represent over three quarters of most instruments, providing a "backbone" of critical assessment information. Instruments applying to individuals who are likely to appear in more than one setting are also coordinated. For example, the majority (68%) of items in the interRAI AC are contained within the interRAI HC; and the interRAI MH contains 84% of the items in the interRAI CMH. Because of frequent movement of individuals between hospital and community settings, this overlap is expected to facilitate a common understanding of the person's needs, and to thus improve continuity of care.

### Interpretation of clinical observations

In addition to the "minimum data sets" of clinical observations, interRAI instruments comprise manuals to support training and a series of algorithms that "interpret" the clinical findings.

The algorithms generate scales that provide severity measures (e.g., the extent of ADL dependency) or diagnostic screeners (e.g., whether a person has dementia). A group of scales were developed for previous generations of instruments [19,20,26-29]. These have been adapted to the new suite, for use across the range of care domains. Numerous scales are currently available encompassing cognition, communication, mood (depression), instrumental and personal ADL, pain and health stability. The concurrent validity of several scales (against comparable scales in widespread use, such as the Mini-Mental State Examination and the Barthel Index) was re-tested in the initial international field work, with good results. The findings will be reported in a future paper. Further scale development and validation is anticipated as the suite comes into wider use.

Clinical assessment protocols (CAPs) are an integral feature of each individual assessment instrument in the suite. CAPs interpret clinical observations in order to identify opportunities to influence clinical and other outcomes. They perform a combination of functions including problem definition (e.g., the person has incontinence) and indicating an opportunity to intervene (e.g., prevention of falls or pressure ulcer, or improvement of ADL function). The method of development utilized by interRAI to develop CAPs comprised several tasks: review of the relevant scientific literature (e.g. strategies that prevent falls in an at risk population); analysis of existing extensive interRAI data sets to identify sub-populations with adverse (or in some cases positive) outcomes across multiple assessment periods; and expert opinion provided by interRAI members and their associates.

In the new suite, the emphasis has been shifted towards identification of problems where there is evidence that the outcome can be influenced. To date, over 40 CAPs have been developed, ranging from clinically oriented problems such as pressure ulcer and pain, to social issues such as abusive behavior and social relationships. A CAP consists of a computer algorithm which identifies persons to whom the protocol applies, and a set of guidelines which assist clinicians to mount an appropriate response. The guideline component is authored by an international panel of relevant domain experts. The CAPs may be used in their own right as a form of clinical guideline, or they may be used in conjunction with existing guidelines. However, the interRAI CAPs have the advantage of being intimately linked to the assessment process.

### Administrative tools

interRAI instruments have proven particularly useful in a variety of administrative areas. Quality indicators developed for nursing homes have been associated with improvement in care standards [9]. Case-mix tools in long term residential care of older persons and community care are used in several nations as a basis for funding. The extensive clinical information derived from interRAI datasets has provided a powerful capacity to compare caseload complexity and service responses between facilities, regions and nations [7,30]. interRAI is currently updating all of these relevant toolsets for application to the integrated suite.

### Implementation issues

The instrument data sets were released during 2005 and have begun to come into active use in several countries. Several multi-setting demonstrations are being established across the world [31].

The availability of an integrated multi-setting clinical information system provides a wide array of opportunities for clinicians, administrators and researchers. However, implementation of such systems may meet substantial challenges.

Necessarily, implementations will be large scale. The establishment of systems across settings is likely to require significant investment by governments. Specific services are often separately administered by different levels of government – national, state, and regional. Even within one level of government, administrative "silos" can develop which attend to each service type (e.g., hospitals and community care). To further complicate matters, the health and social service systems are usually organized in their own administrative silos. Finally, many elements of service systems are often provided by private agencies with varying levels of independence from government administration. The application of a single integrated clinical information system to such a complex jigsaw of services seems formidable.

There are nations and service arrangements where full implementation is potentially feasible. However, it is more likely that an incremental approach will be successful. Administrations operating two or more related services might apply two or three instruments (e.g., acute and post-acute care; community and institutional mental health). Services with relationships to the organizations that implement instruments may see opportunities to improve inter-operability by implementing other instruments from the suite. Ultimately, the choice not to share instrumentation may place an organization at considerable disadvantage. This effect is likely to be particularly pronounced with a fully-integrated suite.

Reluctance to share instrumentation is also evident in the clinical milieu, particularly in multi-disciplinary settings. Each professional group uses its own instrument set to appraise aspects such as cognition, ADL and mood. There is often a division of labor in which each profession attends to aspects of the problem – medical staff to cognition, occupational therapists to functional activities, and nurses to pressure ulcer prevention. The introduction of a "shared" clinical dataset can be perceived to compromise the quality of instrumentation and may threaten professional autonomy.

Conversely, the use of a shared electronic dataset may present an opportunity to improve productivity through reduction in duplication of data collection, particularly if this information is linked to a wider system that brings previous data to the current setting, and offers an opportunity to pass on information efficiently to the subsequent care setting.

interRAI instruments are intimately dependent on computerization, since an integral feature is a suite of complex algorithms that generate scales, CAPs and numerous other administrative by-products. Thus, a major challenge to implementation is access to computers and the need for a degree of computer literacy. Many service settings are not yet ready for this level of sophistication. However, as the benefits of such systems grow, the case for investment in these capabilities becomes increasingly powerful.

interRAI systems are currently involved in research aimed at improving inter-operability of clinical informations sytems, such as the HL7 standards and Snomed CT clinical nomenclature systems. interRAI systems are of particular interest to this area of technical development because of their widespread use, good psychometric properties and holistic approach to data collection. The use of the same items across clinical settings may simplify some of the translational issues when clinical concepts (such as mobility or memory) are shared.

The distribution of personal information across care settings, notably when the care is provided by different agencies, requires careful attention to privacy issues. Protocols are required to ensure that individuals are comfortable with the sharing of information among their caregivers. However, this is a universal concern which is not specific to the interRAI suite.

## Conclusion

The interRAI suite of instruments represents, to our knowledge, the first major effort to provide an integrated health information system with the potential to provide person-centered information that transcends care settings. It offers opportunities to improve continuity of care, from both efficiency and quality of care perspectives. Training of assessors in the interpretation and application of information drawn from a variety of service settings would be simplified. Administrators and planners would be able to compare caseloads and outcomes across settings. When used to its full potential the interRAI suite can substantially enhance the delivery of health care as a ***system ***responding effectively to the needs of vulnerable populations over their life course.

## Competing interests

The authors declare that they have no competing interests.

## Authors' contributions

LG prepared the initial manuscript, and integrated contributions of other authors. All authors made substantial written contributions to the manuscript, reviewed at least 2 revisions, and have given approval to the final version presented here. JM headed the research program that lead to the development of the instrument suite. Other authors actively contributed to the research program and participated in the many research forums described in the manuscript. All authors read and approved the final manuscript.

## Authors' information

Leonard C. Gray. MD. PhD. Professor in Geriatric Medicine, The University of Queensland, c/- Academic Unit in Geriatric Medicine, University Dept of Medicine

Katherine Berg. PhD. PT. Department Chair of Physical Therapy, University of Toronto, Canada, and Adjunct Professor, University of Waterloo, Canada.

Brant E. Fries. PhD. Professor, Health Management and Policy; Research Professor, Institute of Gerontology, School of Medicine, University of Michigan; and Chief, Health Systems Research, Veterans Administration Healthcare System, Ann Arbor, Michigan, USA.

Jean-Claude Henrard. MD. Professor, Research Unit Health and Ageing, Versaille University, Paris-Oust Medical School, Paris, France.

John P. Hirdes. PhD. Professor, Department of Health Studies and Gerontology, University of Waterloo and Scientific Director, Homewood Research Institute, Waterloo, Ontario, Canada.

Knight Steel. MD. Chief, Geriatric Medicine, Hackensack University Medical Center, New Jersey, USA.

John N. Morris. PhD. Co-Director, Institute for Aging Research, and Alfred A and Gilda Slifka Chair in Social Gerontological Research, Hebrew Senior Life, Boston, Massachusetts, USA.

## Pre-publication history

The pre-publication history for this paper can be accessed here:


